# Supplementary material for: MetaQTL: a package of new computational methods for the meta-analysis of QTL mapping experiments
Source: BMC Bioinformatics. 2007 Feb 8;8:49. doi: 10.1186/1471-2105-8-49 (PMC1808479; doi:10.1186/1471-2105-8-49)
Supplement: Additional File 7 — MetaQTL Package : jar file and tutorial. This Zip archive contains both the MetaQTL JAR file and the files of the tutorial. [file 1471-2105-8-49-S7.zip › org.inra.metaqtl/doc/org/metaqtl/Tree.html]

Tree


|  |  |  |
| --- | --- | --- |
| **Overview** \| **Package** \| **Class** \| **Use** \| **Tree** \| **Deprecated** \| **Index** \| **Help** | |  |
| **PREV CLASS**   **NEXT CLASS** | **FRAMES**    **NO FRAMES**     **All Classes** |  |
| SUMMARY: NESTED \| FIELD \| CONSTR \| METHOD | DETAIL: FIELD \| CONSTR \| METHOD |  |


---


## org.metaqtl Class Tree

```
java.lang.Object
  org.metaqtl.Tree
```

---

``` public class Tree extends java.lang.Object ```

This class defines a tree.

---

| **Field Summary** | |
| --- | --- |
| `TreeNode[]` | `nodes`             The nodes of the tree. |
| `TreeNode` | `root`             The root of the tree. |


**Constructor Summary**

`Tree()`

`Tree(TreeNode root)`


| **Method Summary** | |
| --- | --- |
| `TreeNode` | `getLeaf(int i)` |
| `double[]` | `getLeafHeights()` |
| `int` | `getLeafNumber()` |
| `double[]` | `getNodeHeights()` |
| `int` | `getNodeNumber()` |
| `TreeNode` | `getRoot()` |
| `void` | `initNodeIdx()` |
| `void` | `initReverse()` |
| `void` | `updateLeafIdx(int[] nidx)` |

**Methods inherited from class java.lang.Object**

`clone, equals, finalize, getClass, hashCode, notify, notifyAll, toString, wait, wait, wait`

**Field Detail**

### nodes

```
public TreeNode[] nodes
```

:   The nodes of the tree.

---


### root

```
public TreeNode root
```

:   The root of the tree.


**Constructor Detail**

### Tree

```
public Tree()
```

---


### Tree

```
public Tree(TreeNode root)
```

**Parameters:**: `root` -


**Method Detail**

### getLeafNumber

```
public int getLeafNumber()
```

:   **Returns:**

---


### getNodeNumber

```
public int getNodeNumber()
```

:   **Returns:**

---


### getLeafHeights

```
public double[] getLeafHeights()
```

:   **Returns:**

---


### getNodeHeights

```
public double[] getNodeHeights()
```

:   **Returns:**

---


### getRoot

```
public TreeNode getRoot()
```

:   **Returns:**

---


### getLeaf

```
public TreeNode getLeaf(int i)
```

:   **Parameters:**: `i` - **Returns:**

---


### initReverse

```
public void initReverse()
```

---


### initNodeIdx

```
public void initNodeIdx()
```

---


### updateLeafIdx

```
public void updateLeafIdx(int[] nidx)
```

:   **Parameters:**: `qtl_idx` -


---


|  |  |  |
| --- | --- | --- |
| **Overview** \| **Package** \| **Class** \| **Use** \| **Tree** \| **Deprecated** \| **Index** \| **Help** | |  |
| **PREV CLASS**   **NEXT CLASS** | **FRAMES**    **NO FRAMES**     **All Classes** |  |
| SUMMARY: NESTED \| FIELD \| CONSTR \| METHOD | DETAIL: FIELD \| CONSTR \| METHOD |  |


---
